# Aortic VCAM‐1: an early marker of vascular inflammation in collagen‐induced arthritis

**DOI:** 10.1111/jcmm.12790

**Published:** 2016-02-09

**Authors:** Anne Denys, Gaëlle Clavel, Delphine Lemeiter, Olivier Schischmanoff, Marie‐Christophe Boissier, Luca Semerano

**Affiliations:** ^1^ Inserm UMR 1125BobignyFrance; ^2^ Sorbonne Paris Cité ‐ Université Paris 13BobignyFrance; ^3^ Department of Internal MedicineFondation RothschildParisFrance; ^4^ Inserm UMR 978BobignyFrance; ^5^ Assistance Publique – Hôpitaux de Paris (AP‐HP) Groupe hospitalier Avicenne ‐ Jean Verdier – René MuretService de RhumatologieBobignyFrance

**Keywords:** arthritis rheumatoid, atherosclerosis, vasculature, inflammation, collagen‐induced arthritis, mice, hyperlipidic diet

## Abstract

Cardiovascular disease (CVD) is a major cause of morbidity and mortality in rheumatoid arthritis (RA). There are limited experimental data on vascular involvement in arthritis models. To study the link between CVD and inflammation in RA, we developed a model of vascular dysfunction and articular inflammation by collagen‐induced arthritis (CIA) in C57Bl/6 (B6) mice. We studied the expression of vascular inflammatory markers in CIA with and without concomitant hyperlipidic diet (HD). Collagen‐induced arthritis was induced with intradermal injection of chicken type‐II collagen followed by a boost 21 days later. Mice with and without CIA were fed a standard diet or an HD for 12 weeks starting from the day of the boost. Arthritis severity was evaluated with a validated clinical score. Aortic mRNA levels of vascular cell adhesion molecule‐1 (VCAM‐1), inducible nitric oxide synthase (iNOS) and interleukin‐17 were analysed by quantitative RT‐PCR. Vascular cell adhesion molecule‐1 localization in the aortic sinus was determined by immunohistochemistry. Atherosclerotic plaque presence was assessed in aortas. Collagen‐induced arthritis was associated with increased expression of VCAM‐1, independent of diet. VCAM‐1 overexpression was detectable as early as 4 weeks after collagen immunization and persisted after 15 weeks. The HD induced atheroma plaque formation and aortic iNOS expression regardless of CIA. Concomitant CIA and HD had no additive effect on atheroma or VCAM‐1 or iNOS expression. CIA and an HD diet induced a distinct and independent expression of large‐vessel inflammation markers in B6 mice. This model may be relevant for the study of CVD in RA.

## Introduction

Rheumatoid arthritis (RA) is a chronic, systemic inflammatory disease characterized by both articular and extra‐articular manifestations. Cardiovascular disease (CVD) is a major source of morbidity and mortality in RA and other chronic inflammatory rheumatic diseases 1, 2. The magnitude of the risk of CVD in RA might be comparable to that of type 2 diabetes mellitus 3. Overall, the risk of myocardial infarction, congestive heart failure, and death from CVD is 2‐ to 3‐fold greater for RA patients than the general population. The risk of ischaemic heart disease is not increased before the onset of RA symptoms but seems to increase rapidly after the RA diagnosis 4. Nevertheless, the prevalence of traditional cardiovascular risk factors in RA patients is not higher than in the general population. The disease process in RA emerged as an independent risk factor that contributes to the high incidence of cardiovascular events 5, 6.

The early events in atherosclerosis development are mainly increased level of vascular cell adhesion molecule‐1 (VCAM‐1) and altered inducible nitric oxide synthase (iNOS) activity in vascular smooth muscle cells in the artery wall and in immune cells such as macrophages 7, 8, 9. In addition, VCAM‐1 level is increased in fibroblast‐like synoviocytes and endothelial cells (ECs) from RA synovium 10, 11. Vascular cell adhesion molecule‐1 is mainly involved in leucocyte transendothelial migration and leucocyte retention in the inflamed joint. Hence, common pathways might be responsible for both articular and vascular damage in RA. The inflammatory response in RA may be directly involved in altered EC function, vascular dysfunction and atherosclerosis initiation and progression. Thus, the global cardiovascular burden in RA results from both metabolic factors, such as dyslipidemia, and inflammation.

To explore the link between joint inflammation, vascular dysfunction and cardiovascular risk in RA, we need animal models that can associate articular and systemic inflammation with vascular dysfunction. Atherosclerosis does not develop spontaneously in mice, except when they are fed a hyperlipidic diet (HD) or are genetically modified or one of the carotids is injured. Apolipoprotein E knockout (ApoE^−/−^) mice, a model of atherosclerosis, are sensitive to collagen‐induced arthritis (CIA). Nevertheless, despite a documented expansion of pro‐inflammatory T‐helper 1 cells (Th1) and Th17 cells, arthritis does not affect atherosclerosis severity in such mice 12, 13. K/BXN mice, a spontaneous arthritis model, can show atherosclerotic aortic plaques independent of diet, and etanercept treatment can prevent plaque formation 14. In DBA/1 (H2‐q) mice, which are resistant to atherosclerosis development, collagen immunization resulted in loss of vascular elasticity and altered endothelial nitric oxide signalling 15.

In this study, we used C57Bl/6 (B6) (H2‐b) mice immunized with chicken collagen type II (cCII) and complete Freund's adjuvant (CFA) to investigate whether the pro‐inflammatory context associated with arthritis development (CIA) induced large‐vessel dysfunction. Moreover, we assessed the effect of HD in the same model. Finally, we studied modifications in large‐vessel molecules and atheroma formation induced by concomitant cCII immunization and HD to dissect their respective roles in inducing vascular damage.

## Materials and methods

### Arthritis induction

Arthritis was induced by use of native cCII (Morwell Diagnostics, Zurich, Switzerland). Male C57Bl/6 JRJ (B6) mice (Janvier, France), 11 weeks old, were immunized with cCII (100 μg)/CFA (2 mg/ml) by intradermal injection, then received a boost [cCIIp (100 μg)/CFA (2 mg/ml)] 21 days later. We assessed arthritis development in hind paws and forepaws with clinical scores ranging from 0 (no evidence of erythema and swelling) to 4 (erythema and severe swelling encompassing the ankle, foot and digits). The mice were monitored until arthritis scores started to decrease. All procedures were approved by the Animal Care and Use Committee of the University of Paris 13 (ethical approval ID: Ce5/2010/037).

### Anti‐CII antibody assay

Anti‐collagen IgG antibodies were evaluated by ELISA in B6 mice. After cCII adsorption on microtitration plates, mice sera, diluted at 1/1000 was incubated on coated plates. Anti‐cCII IgG antibodies were detected with alkaline phosphatase‐linked rat anti‐mouse IgG antibody (Sigma‐Aldrich, Saint‐Quentin Fallavier, France) after incubation with its para‐nitrophenylphosphate substrate (Sigma‐Aldrich) and read at 405 nm. Sera of CIA mice with severe disease from previous experiments was used as a positive control.

### Diet and lipid measurement

Mice were fed a high cholesterol (HD) diet (Safe, Augy, France), which was pro‐atherogenic 16, or standard chow diet (Harlan, Gannat, France) for 12 weeks starting from the day of the second immunization boost (D21). Blood was collected by cardiac puncture. Levels of total cholesterol, HDL cholesterol and triglycerides (TG) in serum were measured by an enzymatic method on a Cobas CEC system (Roche Diagnostic System). Low‐density lipoproteins (LDL) cholesterol level was calculated by the standard Friedewald equation 17.

### Staining of atherosclerotic aortic plaque

Mice were killed and aortas were removed 15 weeks after the first collagen immunization, washed in cold water, then incubated in propan‐2‐ol (60%) for 30 sec. Tissue was incubated in Oil‐red O (0.24%) as per the manufacturer's protocol (Sigma‐Aldrich) in propan‐2‐ol for 18 min., then in propan‐2‐ol for 30 sec. After a washing in water, tissue was covered with cover slips fixed with aqueous mounting medium and photographed by use of a binocular loupe (magnification ×16) and camera (Nikon Coolpix S9300, Nikon, Tokyo, Japan).

### Quantification of synovial and aortic pro‐inflammatory molecules by use of quantitative real time polymerase chain reaction

The expression of pro‐inflammatory molecules was checked in aortas excised 15 weeks after the first collagen immunization. In addition, both early and late vascular modifications associated with collagen immunization were studied in synovium and aortas in mice killed at 4 and 15 weeks after the first administration of cCII/CFA emulsion or CFA without cCII. Synovial and aortic tissues were collected in Lysing matrix D bulk containing 1.4 mm ceramic spheres (Lysing Matrix D tube RNAase/DNAase free MP Biomedicals) with1 ml Tri‐reagent (synovium) or 1 ml Qiazol (aorta) added for total RNA extraction (Euromedex, Souffelweyersheim, France) in accordance to the manufacturer's instructions. Then, samples were mixed with use of a Pulverizer System (MP Biomedical Fastprep 24). Chloroform was then added. RNA was taken from the uppermost aqueous phase and precipitated with isopropanol. Ethanol was added to wash out the RNA, which was then diluted in a final volume of 20 μl of H_2_O diethyl pyrocarbonate. Total RNA was used to synthesize cDNA by use of SuperscriptIII RNase H‐reverse transcriptase (RT; Invitrogen, Paris, France) in a total volume of 20 μl. PCR reactions were performed in a volume of 15 μl containing oligonucleotide primers (0.4 μM; Table 1) for the reporter gene actin, mouse primer for analysed molecules [interleukin 17 (IL‐17), VCAM‐1, iNOS] and Fast start DNA master plus SYBR Green (Roche Diagnostics, Meylan, France) containing Taq polymerase dNTP, reaction buffer, and the double‐stranded DNAspecific fluorescent dye SYBR Green I. DNA was amplified with use of LightCycler (Roche Applied Science, Penzberg, Germany). The reaction cycle was carried out using the following thermal cycling programme: cytokine denaturation step at 95°C for 8 min., 45 denaturation cycles at 95°C for 10 sec., annealing at 62°C for 5 sec., and elongation at 72°C for 8 sec.; the actin step consisted of denaturation at 95°C for 8 min. then 45 denaturation cycles at 95°C for 10 sec., annealing at 62°C for 5 sec., and elongation at 72°C for 8 sec. The fluorescent signal was picked up at the end of the elongation step. Relative cytokine transcript levels were calculated by use of RealQuant software (Roche Biochemicals) and expressed in arbitrary units.

**Table 1:** Primers used in qRT‐PCR (Universal probe library, Roche)

Gene	Sequences (5′–3′)
Actin (FW)	AGAGGGAAATCGTGCGTGAC
Actin (RV)	CAATAGTGATGACCTGGCCGT
VCAM‐1 (FW)	GCTATGAGGATGGAAGACTCTGG
VCAM‐1 (RV)	ACTTGTGCAGCCACCTGAGATC
IL‐17 (FW)	AGCTGGACCACCACATGAA
IL‐17 (RV)	AAACGTGGGGGTTTCTTAGG
iNOS (FW)	ATCCAGTGCCCTGCTTCA
iNOS (RV)	GCAGGGCAAGTTAGGATCAG

FW: forward; RV: reverse.

### Immunohistochemistry

Immunohistochemistry involved use of monoclonal antibody clone 429 to purified VCAM‐1 (10 μg/ml). Frozen tissue sections were thawed and fixed in acetone for 5 min., dried, and rehydrated in PBS. The primary antibody or the corresponding isotype control (eBiosciences) was applied at the indicated dilutions in 0.5% bovine serum albumin (BSA) in PBS and incubated in a humidified chamber for 60 min. at room temperature. Sections were washed in PBS, and then incubated with a biotinylated secondary antibody (horse anti‐rat IgG; eBiosciences) in PBS containing 0.5% BSA for 30 min. at room temperature. Then, sections were washed in PBS and incubated with the avidin‐biotin enzyme complex (Vecstatin ABC complex) and chromogenic substrate (DAB) as per the manufacturer's instruction. All sections were counterstained with haematoxylin and lithium carbonate. Quantitative analysis of VCAM‐1–positive staining in the aortic sinus involved use of Image J Fiji.

### Statistical analysis

Data were compared by parametric (anova, Student's *t*‐test) or nonparametric (Kruskal–Wallis, Mann–Withney) tests, according to the data distribution, with appropriate *post hoc* comparisons. Statistical analyses involved use of MedCalc v10.4 (MedCalc Software bvba, Mariakerke, Belgium). *P* < 0.05 was considered statistically significant.

## Results

### Arthritis development was not affected by an HD while collagen immunization did not affect plaque formation in mice

**Figure 1:**
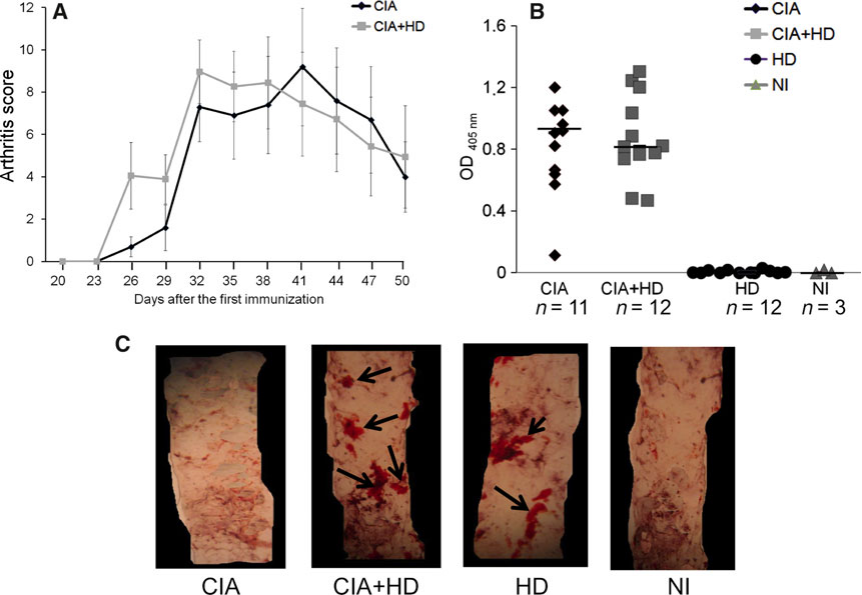
Effect of hypercholesterolemic diet (HD) on collagen‐induced arthritis (CIA), anti‐collagen antibody production and aortic lipid accumulation in mice. B6 mice were immunized with two injections of chicken CII (cCII). HD was started from the day of the second immunization (day 21). (**A**) Arthritis scores in immunized mice receiving HD (CIA+HD) (*n* = 12) or standard chow diet (CIA alone) (*n* = 12). Data are mean ± S.E.M. (**B**) Anti‐cCII antibody levels detected in mice at 15 weeks after the first immunization in CIA, CIA+HD, nonimmunized (NI) mice fed an HD or a standard chow diet. Horizontal bar is median Ab level. (**C**) Aortic lipid accumulation detected on thoracic sections by Oil‐red O staining (magnification ×16).

We analysed arthritis development and atherosclerosis in CIA mice fed an HD or standard diet. Arthritis development was similar in both groups of mice (Fig. 1A). The onset of clinical arthritis was at day 27 for both groups; a decrease in clinical score was evident at day 50. The severity of the disease did not significantly differ. All immunized mice had high of anti‐cCII antibodies, regardless of diet (Fig. 1B). In aortas removed from mice at 15 weeks after the first cCII immunization (i.e. after 12 weeks of HD for CIA+HD mice), atherosclerotic plaque formation was detected with CIA+HD and HD but not CIA alone (Fig. 1C). Therefore, HD did not affect CIA development in immunized mice and CIA did not affect atherosclerotic plaque formation in aortas of mice fed an HD.

### Dyslipidemia was observed in mice fed an HD independent of collagen immunization

Serum lipid profile was quantified 15 weeks after the first immunization. HD had been administered for 12 weeks to both a group of immunized (CIA+HD) and a group of nonimmunized (HD) mice. Both CIA+HD and HD mice had significantly higher levels of total cholesterol, HDL‐C and LDL‐C compared to mice receiving a standard diet, whether immunized (CIA) or nonimmunized (NI; *P* < 0.05; Table 2). CIA+HD mice had lower TG than CIA, HD and NI mice (*P* < 0.05). Levels of total cholesterol, HDL‐C, LDL‐C and TG in mice fed a standard diet did not differ with and without immunization (CIA and NI; Table 2).

**Table 2:** Body weight and plasma lipid levels of mice with various treatments

Treatment	Body weight (g)	Total cholesterol level (mM)	HDL‐C level (mM)	LDL‐C level (mM)	TG level (mM)
CIA	27.1 ± 0.4	2.61 ± 0.37	2.3 ± 0.33	0.2 ± 0.09	0.53 ± 0.14
CIA+HD	28.4 ± 0.5	3.78 ± 0.12^a^	3.03 ± 0.13^a^	0.59 ± 0.06^a^	0.25 ± 0.02^b^
HD	27.8 ± 0.4	4.2 ± 0.1^a^	3.59 ± 0.09^a^	0.51 ± 0.05^a^	0.5 ± 0.04
NI	30.01 ± 0.5	2.59 ± 0.17	2.28 ± 0.15	0.19 ± 0.15	0.61 ± 0.22

a *P* < 0.05 *versus* CIA alone and NI.

b *P* < 0.05 *versus* CIA, HD and NI alone (Kruskall–Wallis).

Data are mean ± S.E.M.

CIA: collagen‐induced arthritis; HD: hyperlipidic diet; NI: nonimmunized; HDL‐C: high‐density lipoprotein cholesterol; LDL‐C: low‐density lipoprotein cholesterol; TG: triglycerides.

### mRNA levels of pro‐inflammatory molecules in aorta and synovial membrane of mice with CIA

**Figure 2:**
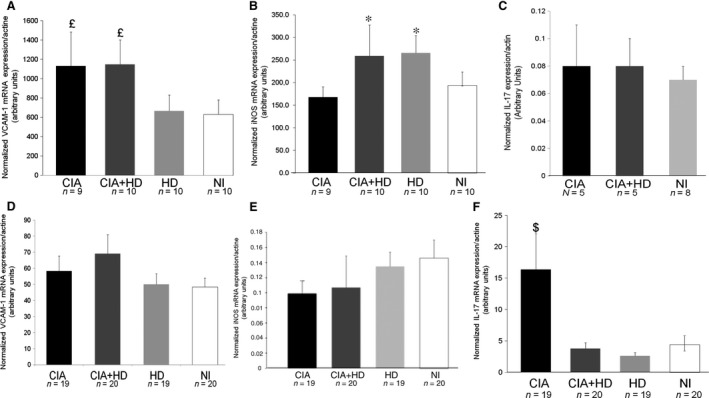
mRNA expression of pro‐inflammatory molecules in aortas (**A**–**C**) and synovium (**D**–**F**) of immunized and nonimmunized mice fed an HD or standard chow diet. Quantitative RT‐PCR (qRT‐PCR) analysis of mRNA levels of vascular cell adhesion molecule‐1 (VCAM‐1) (**A** and **D**), inducible nitric oxide synthase (iNOS) (**B** and **E**) and interleukin 17 (IL‐17) (**C** and **F**). Data are mean ± S.E.M. ^£^ *P* < 0.05 *versus* HD and NI; *P < 0.05 *versus* CIA and NI, ^$^ *P* < 0.05 *versus* CIA+HD, HD and NI.

We evaluated the mRNA expression of pro‐inflammatory molecules known to be involved in arthritis and atherosclerosis development. In synovial membranes and aortas collected at 15 weeks after the first collagen immunization, VCAM‐1 mRNA level was increased in aortas from both CIA and CIA+HD as compared with HD and NI (Fig. 2A; *P* < 0.05). Vascular cell adhesion molecule‐1 mRNA expression did not differ between CIA and CIA+HD. This suggests that VCAM‐1 level in immunized mice may not be affected by diet. Conversely, iNOS mRNA expression was higher with the HD (both CIA+HD and HD) than CIA and NI alone (*P* < 0.05; Fig. 2B). Therefore, iNOS expression depends on the HD and is not affected by CIA. The aortic mRNA level of IL‐17 (Fig. 2C) and IL‐6 (data not shown) did not differ by treatment. In synovium, VCAM‐1 and iNOS mRNA levels did not differ among groups (Fig. 2D, E). Nevertheless, IL‐17 level was significantly higher with CIA and a standard diet than with other treatments (Fig. 2F; *P* < 0.05).

### Early aortic VCAM‐1 mRNA overexpression in CIA

**Figure 3:**
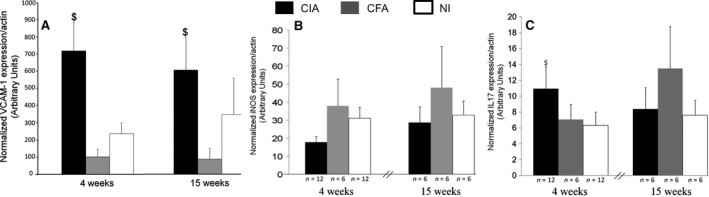
Early (4 weeks) and late (15 weeks) aortic mRNA expression of VCAM‐1, iNOS, and IL‐17 in CIA mice. Aortas from CIA mice were removed at 4 weeks (*n* = 12) and 15 weeks (*n* = 6) after immunization. Controls were: mice immunized with only CFA (*n* = 6) and nonimmunized mice (NI) (*n* = 12 or *n* = 6 for aorta removed at 4 or 15 weeks, respectively). qRT‐PCR analysis of mRNA levels of VCAM‐1 (**A**), iNOS (**B**) and IL‐17 (**C**). Data are mean ± S.E.M.; ^$^ *P* < 0.01 *versus* CFA and NI; ^£^ *P* < 0.05 *versus* CFA.

After showing VCAM‐1 overexpression in aorta from CIA mice 15 weeks after the first collagen immunization, we evaluated whether this CIA‐associated vascular dysfunction could be detected earlier (i.e. 4 weeks after the first collagen immunization). As arthritis was induced with the cCII‐CFA emulsion, we checked whether CFA immunization alone resulted in arthritis or aortic inflammation (Fig. 3). Immunization with cCII‐CFA (CIA) emulsion induced arthritis, with 70% to 85% incidence, and production of anti‐cCII antibodies. As expected, mice immunized with CFA alone did not show arthritis and did not produce anti‐cCII antibodies (data not shown). At 4 weeks after the first immunization, VCAM‐1 mRNA expression was significantly increased in aortas from CIA mice as compared with both CFA and NI mice (*P* < 0.01; Fig. 3A). This difference remained significant at 15 weeks after the first immunization as compared with CFA alone (*P* < 0.05), which confirmed the results shown in Figure 2A. Conversely, aortic iNOS expression was not significantly altered with CIA as compared with CFA and NI (Fig. 3B). Aortic IL‐17 level was significantly increased with CIA at 4 weeks after the first immunization (*P* < 0.05; Fig. 3C) but not at week 15.

**Figure 4:**
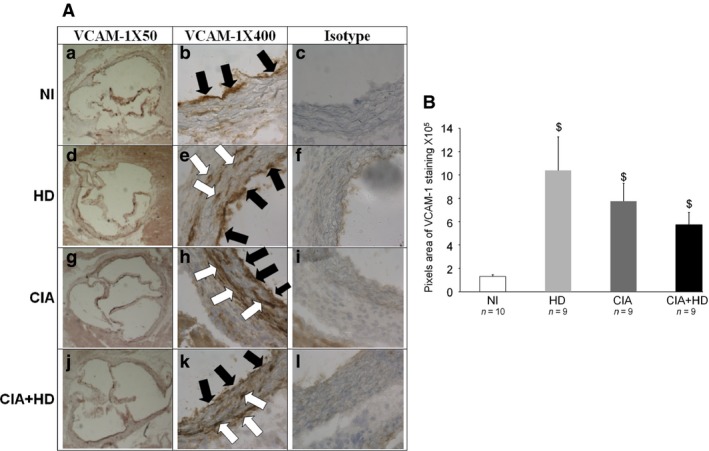
VCAM‐1 staining in aortic sinus from mice fed an HD or standard chow diet. (**A**) Representative histological sections of the aortic sinus stained with blue‐haematoxylin for VCAM‐1 in NI mice fed a standard chow diet or an HD and in immunized mice fed a standard diet (CIA) or an HD (CIA+HD). Magnification is 50 × (a, d, g, j) or 400 × (b, e, h, k). Staining for isotype control of VCAM‐1 antibody (IgG2a, κ antibody) is shown in c, f, i and l (×400 magnification). Black arrows show VCAM‐1 endothelial staining; white arrows show VCAM‐1 staining in the aortic sinus wall. (**B**) Quantification of VCAM‐1–positive staining by use of Image J Fiji. ^$^ *P* < 0.05 *versus* NI.

### Detection of VCAM‐1 protein in the aortic sinus in CIA

After finding increased VCAM‐1 expression in mouse aortas with CIA and CIA+HD, we localized VCAM‐1 in the aortic sinus by immunohistochemistry. The aortic sinus is a dilatation between the aortic wall and each cusp of the aortic valve. It is limited by the endothelial surface in contact with turbulent blood flow, which induces low shear stress 18. Staining for VCAM‐1 for NI mice was limited to the endothelial surface (Fig. 4A). Conversely, with CIA, HD alone or both, VCAM‐1 expression was localized on both the endothelial surface and the aortic sinus wall. The control isotype was negative for each sample. Quantification of VCAM‐1 showed significantly higher staining with CIA, CIA+HD and HD *versus* NI mice (*P* < 0.05; Fig. 4B).

### Staining for VCAM‐1 in the aortic sinus from immunized mice with CIA increases over time

**Figure 5:**
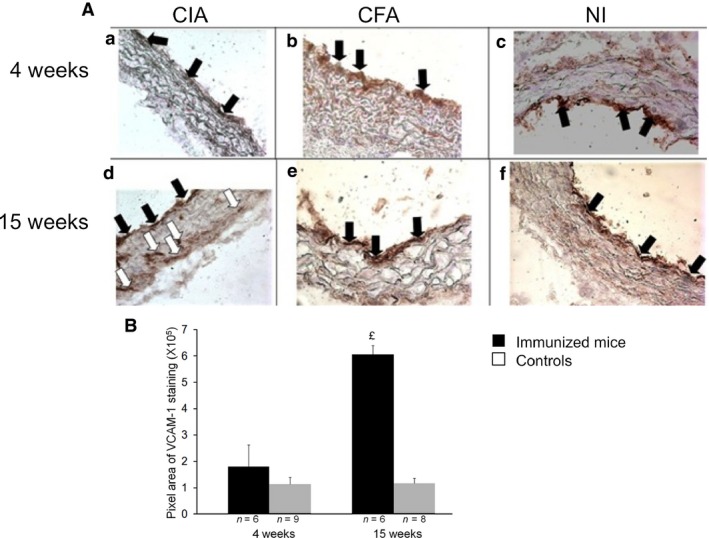
Early (4 weeks) and late (15 weeks) VCAM‐1 staining in aortic sinus of mice. Aortic sinuses from CIA mice were removed at 4 and 15 weeks after immunization. Controls were immunized with only CFA or were NI mice. (**A**) Representative microphotographs of VCAM‐1 staining in aortic sinus from cCII/CFA‐immunized (CIA: a, d), CFA‐immunized (CFA: b, e) and NI(c, f) mice. Black arrows show endothelial staining for VCAM‐1; white arrows show VCAM‐1 staining in the aortic sinus wall. (**B**) Quantification of VCAM‐1–staining at 4 and 15 weeks in CIA *versus* control mice (NI and CFA mice pooled) by use of Image J Fiji. ^£^ *P* < 0.05 *versus* CT.

At 4 weeks after the first collagen immunization, VCAM‐1 staining was limited to the endothelial surface of the aortic sinus with CIA, in mice treated with CFA and in NI mice (Fig. 5A, a, b, and c). At week 15, VCAM‐1 expression was detected on both the endothelial surface and inside the aortic sinus wall with CIA (Fig. 5A, d) but not in CFA and NI mice (Fig. 5A, e, f). The isotype control was negative for each sample (data not shown). Vascular cell adhesion molecule‐1 staining was greater with CIA *versus* control mice (a pool of NI and CFA mice; *P* < 0.05; Fig. 5B).

## Discussion

In this study, immunizating B6 mice with cCII and CFA to produce CIA induced vascular dysfunction in large blood vessels, notably the precocious expression of the adhesion molecule VCAM‐1. In the same model, HD induced atheroma plaque formation and increased iNOS expression. Thus, experimental arthritis and a lipid‐enriched diet have different and independent effects on large vessels. This finding is confirmed by the lack of amplified vascular dysfunction in mice with CIA fed an HD.

A major challenge of this study was to identify a mouse strain susceptible to both experimental arthritis and atherosclerosis. DBA/1 mice are highly susceptible to cCII immunization but are resistant to atherosclerosis development 19, 20. B6 mice show atherosclerotic aortic plaques after 12 weeks of HD 19, 20. Therefore, we focused on developing CIA in B6 mice. This animal model allowed us to establish large‐vessel dysfunction (i.e. VCAM‐1 overexpression) as early as 4 weeks after the first collagen immunization.

Our results suggest that the pro‐inflammatory milieu caused by arthritis induction might initiate vascular dysfunction in large blood vessels. CIA drives the expression of pro‐inflammatory molecules such as tumour necrosis factor‐α, IL‐1 and IL‐6 21. These cytokines exert their action at both local and systemic level 22, 23, 24, thereby playing a role in vascular damage 11. Vascular smooth muscle cells and ECs are the two main cell types involved in vascular homoeostasis 25, 26. The inflammatory environment activates VCAM‐1 expression on ECs, thereby allowing for leucocyte adhesion and infiltration in inflamed tissue 27, 28. In our study, VCAM‐1 mRNA expression in the mouse aorta was precociously increased with cCII immunization. This suggests that cCII immunization triggers an inflammatory reaction that is not confined to joints, but has rather a systemic character, involving large vessels far from articular sites. Large vessels inflammation would then reflect into early, but long‐lasting increased expression of endothelial adhesion molecules like VCAM‐1.

Unlike qRT‐PCR, that was performed on the whole aorta, we could perform immunostaining only in the aortic sinus. In the aortic sinus, VCAM‐1 is constitutively expressed on the endothelial surface 29. At 4 weeks after cCII immunization of mice, aortic‐sinus VCAM‐1 staining did not differ between CIA and control mice but was greater in CIA mice at 15 weeks. Nevertheless, VCAM‐1 overexpression in the sinus is not specific to CIA, because both CIA and HD increased VCAM‐1 aortic‐sinus staining. The sinus is currently used in atherosclerosis models to quantify lesions and evaluate anti‐atheromatous therapeutic strategies 11, 12, 29, 30, 31, 32. These results suggest that the aortic sinus is likely sensitive to both metabolic (high fat diet) and inflammatory cues. Thus, aortic‐sinus staining for VCAM‐1 is less useful to distinguish early, specific vascular injuries associated with CIA.

Vascular cell adhesion molecule‐1 is also expressed on several cell types in the RA synovium 33, 34, and preliminary treatment of mice with anti‐VCAM‐1 antibody reduced joint involvement in CIA 34. We analysed VCAM‐1 expression in the mouse synovium at 4 and 15 weeks after cCII immunization but found no difference in VCAM‐1 expression between CIA mice and controls.

Serum levels of total cholesterol, LDL‐C and HDL‐C were higher in both immunized and NI mice fed an HD than those fed a standard diet starting from the day of the CIA boost. Immunized mice fed a HD showed high serum cholesterol but significantly lower serum TG, a phenomenon already described in inflammatory models 35. Interestingly, in mice lacking the liver‐X‐receptor (LXR) fed a high‐cholesterol diet, serum TG are dramatically reduced because of hepatic accumulation of cholesteryl esters that compete with TG for incorporation into very low‐density lipoproteins (VLDL) 36. As inflammation may reduce the activity of LXR 37, immunized mice fed a HD may also be less capable to incorporate TG into VLDL.

All HD‐fed mice showed atherosclerotic aortic plaques and increased iNOS mRNA expression in the aorta, which suggests that in this model, iNOS expression is specific to HD rather than to systemic inflammation.

Nitric oxide may also play a role in the pathogenesis of synovitis and bone destruction 37, 38, 39, 40 and some studies showed increased expression of iNOS in synovial cells and oxidized nitric oxide levels in synovial fluid in RA 40, 41, 42. However, we detected no significant difference in iNOS expression in synovium between CIA, CFA and NI mice.

Finally, Th17 has been found involved in arthritis development in CIA, but its role in vascular dysfunction in this context has not been described 43, 44. Moreover, its role in vascular dysfunction such as atherosclerosis is controversial 45, 46, 47. According to Madhur *et al*., IL‐17A is necessary to induce superoxide vascular production in response to a high fat diet in ApoE^−/−^ mice 48. In our study, neither CIA nor HD had any effect on IL‐17 mRNA expression in the mouse aorta. Quite surprisingly, immunized mice receiving concomitant HD showed lower synovial IL‐17 expression *versus* immunized mice fed a chow diet. In our preliminary experiences we found that in CIA, HD given before arthritis induction did prevent arthritis. Nevertheless, in the present work, HD was given after arthritis onset and did not interfere with arthritis development (Fig. 1).

In conclusion, we show that CIA and HD induced a different profile of aortic inflammation in B6 mice: HD produced high iNOS expression, whereas CIA produced VCAM‐1 overexpression. Thus, in B6 mice, HD and CIA induced a distinct and independent expression of large‐vessel inflammation markers. As VCAM‐1 specifically reflects large‐vessel inflammation induced by arthritis, this early inflammatory marker may be relevant for evaluating the impact of anti‐arthritic therapies on vascular damage.

## Conflicts of interest

The authors declare that they have no competing financial or nonfinancial interests in relation to this manuscript.
